# Differences in characteristics and infection severity between odontogenic and other bacterial oro-naso-pharyngeal infections

**DOI:** 10.1186/s13005-023-00354-5

**Published:** 2023-03-15

**Authors:** Suvi-Tuuli Vilén, Hanna Ahde, Tuukka Puolakka, Antti Mäkitie, Johanna Uittamo, Johanna Snäll

**Affiliations:** 1 Department of Oral and Maxillofacial Diseases, University of Helsinki, Haartmaninkatu 1, PL 220, 00029 Helsinki, HUS Finland; 2 Department of Emergency Medicine & Services, Helsinki University Hospital and University of Helsinki, Helsinki, Finland; 3 Department of Otorhinolaryngology-Head and Neck Surgery and Research Program in Systems Oncology, Helsinki University Hospital and University of Helsinki, Helsinki, Finland; 4 Department of Oral and Maxillofacial Diseases, Helsinki University Hospital and University of Helsinki, Helsinki, Finland

**Keywords:** Odontogenic infection, Oro-naso-pharyngeal infection, Intensive care unit, Hospital stay

## Abstract

**Background:**

Different bacterial infections of the oro-naso-pharyngeal (ONP) region may progress and require hospital care. The present study clarified differences in infection characteristics between hospitalized patients with odontogenic infections (OIs) and other bacterial ONP infections. The specific aim was to evaluate clinical infection variables and infection severity according to infection aetiology, particularly regarding features of OIs compared with other ONPs.

**Methods:**

Records of patients aged ≥16 years requiring hospital care for an acute bacterial ONP infection in the emergency units of Otorhinolaryngology or Oral and Maxillofacial Surgery at the Helsinki University Hospital (Helsinki, Finland) during 2019 were evaluated retrospectively. The main outcome variables were need for intensive care unit (ICU) treatment and length of hospital stay. The primary predictor variable was infection category, defined as OI or other ONP. The secondary predictor variable was specific ONP infection group. Additional predictor variables were primary clinical infection signs, infection parameters at hospital admission, and delay from beginning of symptoms to hospitalization. Explanatory variables were sex, age, current smoking, heavy alcohol use or substance abuse, and immunosuppressive disease, immunosuppressive medication, or both. Comparison of study groups was performed using Fisher’s exact test, student’s *t*-test, and Mann-Whitney *U*.

**Results:**

A total of 415 patients with bacterial ONPs fulfilled the inclusion criteria. The most common infections were oropharyngeal (including peritonsillar, tonsillar, and parapharyngeal infections; 51%) followed by infections from the odontogenic origin (24%). Clinical features of OIs differed from other ONPs. Restricted mouth opening, skin redness, or facial or neck swelling (or both) were found significantly more often in OIs (*p* < 0.001). OIs required ICU care significantly more often than other ONPs (*p* < 0.001) and their hospital stay was longer (*p* = 0.017).

**Conclusions:**

Infections originating from the tonsillary and dental origin had the greatest need for hospitalization. Clinical features of OIs differed; the need for ICU treatment was more common and hospital stay was longer compared with other ONPs. Preventive care should be emphasized regarding OIs, and typical infection characteristics of ONP infection subgroups should be highlighted to achieve early and prompt diagnosis and treatment and to reduce hospitalization time.

## Background

Bacterial infections of the oro-naso-pharyngeal (ONP) region are common and usually mild. However, these infections may also progress to more serious disease when the infection spreads to deep tissues or the general condition of patient requires hospitalization [1, 2]. ONP infections with bacterial aetiology that require hospitalization originate from different sources. Deep-neck infections most often originate from either odontogenic (29–71%) [1–6] or tonsillar/peritonsillar focus (16–30%) [1, 2, 4, 6]. Infections of major salivary glands account for 12–19% of severe ONP infections [5, 7, 8]; epiglottitis accounts for 6% [5, 7]. Other infectious disorders in the ONP region, such as trauma-related and iatrogenic infections, infected cysts, or tumours may lead to hospitalization [1, 2, 5, 6]. A typical feature of ONP infections is that the initial origin remains unknown in some patients [1, 2, 5, 6].

In general, the most complex ONP infections are related to alcohol abuse, immunosuppression, psychiatric disorders [9], immunosuppressive diseases, and specific clinical features [9, 10]. On the other hand, patients with deep odontogenic abscesses are typically previously healthy without significant immunosuppressive diseases [11]. Differences in infection spread and severity between ONP infection subtypes, and especially the features of odontogenic infections (OI) compared with other ONP infections, have been seldom clarified. Staffieri et al. reported a shorter hospital stay for OI patients than patients with other infection focus [12]. In turn, deep OIs more likely required repeated surgery than other severe ONP infections [1]. Nonetheless, OIs do not increase complication risk [12]. However, the inclusion criteria and settings of these studies varied considerably and earlier studies of ONP infections focused mainly on the most severe deep-neck infections [1, 2, 4, 7, 8]. Limited results have been presented on healthcare burden.

We decided to clarify the characteristics of all hospitalized ONP patients and in particular the features of OIs compared with other ONP infections. The purpose of the present study was to clarify differences in infection characteristics between patients with hospitalized OIs and other bacterial ONP infections. We hypothesized that OIs have special features which differ from other infections of the same region.

## Methods

### Study design and inclusion criteria

Data of all hospitalized patients with an acute infection diagnosed at the Oral and Maxillofacial Surgery Trauma Center or Otorhinolaryngology – Head and Neck Surgery Emergency Departments of the Helsinki University Hospital, Helsinki, Finland between 1 January and 31 December 2019 were included. These departments have a catchment area of approximately 1.6 million inhabitants.

Patient data were extracted from electronic patient records by diagnosis. Included were hospitalized patients who were treated for acute bacterial odontogenic, other oropharyngeal, or sinus infection. Patients with unclear infection, cutaneous infection, or solely virus infection were excluded.

### Study variables

The main outcome variables were need for intensive care unit (ICU) treatment and duration of hospital stay.

The primary predictor variable was infection category defined as OI or other ONP infection. Secondary predictor variable was specific infection group categorized as OI, peritonsillar or tonsillar infection or parapharyngeal infection (or combinations thereof), sinusitis, epiglottitis, or supraglottitis (or combinations thereof), or sialadenitis.

Additional predictor variables were primary clinical infection signs, infection parameters at hospital admission, and delay from beginning of symptoms to hospitalization.

The explanatory variables were sex, age, current smoking, heavy alcohol use or substance abuse, and immunosuppressive disease, immunosuppressive medication, or both. Limits for heavy alcohol use were ≥ 12 doses (i.e. ≥150 g alcohol) per week for women and ≥ 23 doses (i.e. ≥287.5 g of alcohol) per week for men.

Duration of ICU stay and duration of hospital stay were also reported.

### Statistical analysis

Comparison of study groups was done using Fisher’s exact test, student’s *t*-test, and Mann-Whitney *U* where appropriate. Binary logistic regression was used to determine the association between selected variables and need for ICU stay. Significance was set at *p* < 0.05. Statistical analysis was performed using SPSS Statistics 25 software (IBM).

## Results

Records of 688 patients were evaluated for the study. Of these, 273 were excluded for virus infection or unknown aetiology. In all, 415 patients with bacterial ONPs fulfilled the inclusion criteria and were included for the final analyses. Patient age ranged between 16.1 and 95.1 years (mean 21.7, median 44.1).

**Fig. 1:**
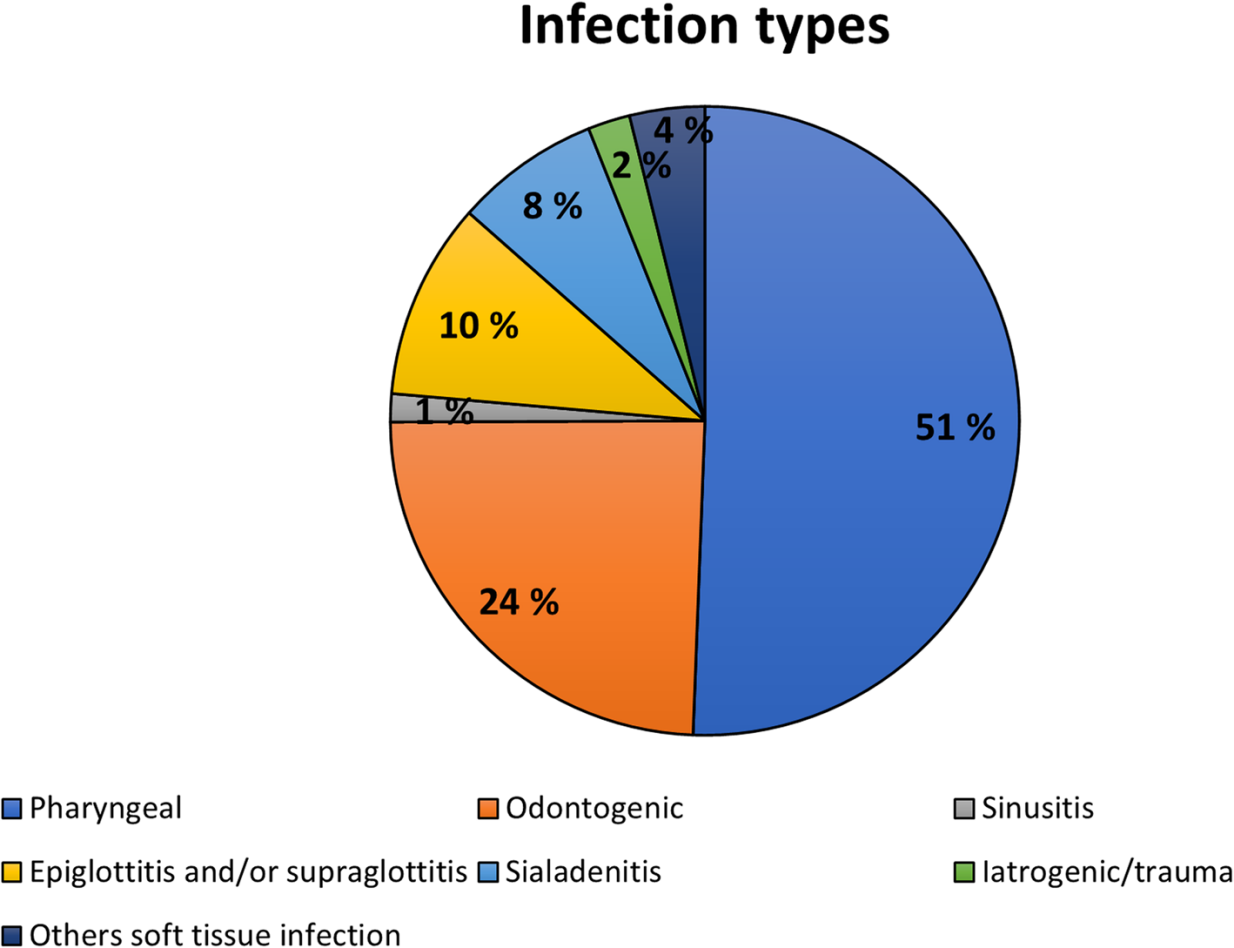
Oro-naso-pharyngeal infection types. The most common infection types were oropharyngeal infections followed by infections of dental origin

The most common infections were oropharyngeal, including peritonsillar, tonsillar, and parapharyngeal infections (51%) followed by infections of odontogenic origin (24%). The remaining categories were less common. Epiglottitis, supraglottitis, or both were found in 10% and sialadenitis in 7.5% of the patients (Fig. 1).

Patients with OI were significantly older than patients with ONPs (*p* = 0.010) and were more often immunocompromised (*p* = 0.017) (Table 1). Clinical features of OIs differed from ONPs. Restricted mouth opening, skin redness, and facial or neck swelling (or both) were observed significantly more often in OIs (*p* < 0.001), although dysphagia was more common in ONPs (*p* < 0.001). Patients with OIs also more often had fever than patients with ONPs (*p* < 0.001). Of patients with OIs, 96.0% received surgical treatment; the corresponding proportion was 61.1% for ONP patients (*p* < 0.001).

**Table 1:** Associations between background variables and infections of odontogenic and non-odontogenic aetiology

	Patients with odontogenic infection			Patients without odontogenic infection			
	n	%	% of patients with odontogenic infection	n	%	% of patients without odontogenic infection	***p-***value
**All**	101	24.3		314	75.7		
**Sex**							
Male	60	59.4	24.7	183	58.3	75.3	
Female	41	40.6	23.8	131	41.7	76.2	^a^*p* = 0.908
**Age (years)**							
Range	18–89			16–95			
Mean	48.5			42.4			
Median	50.8			38.5			^b^*p* = 0.010
**Smoking**							
Yes	24	23.8	25.3	71	22.6	74.7	
No	35	34.7	25.0	105	33.4	75.0	
Not known	42	41.6	23.3	138	43.9	76.7	^a^*p* = 1.000
**Immunodeficiency**							
Yes	24	23.8	36.9	41	13.1	63.1	
No	77	76.2	22.0	273	86.9	78.0	^a^*p* = 0.017
**Swallowing difficulty**							
Yes	38	37.6	15.1	213	67.8	84.9	^a^*p* < 0.001
No	61	60.4	39.1	95	30.3	60.9	
Not known	2	2.0	25.0	6	1.9	75.0	
**Restricted mouth opening**							
Yes	69	68.3	44.5	86	27.4	55.5	^a^*p* < 0.001
No	30	29.7	17.0	146	46.5	83.0	
Not known	2	2.0	2.4	82	26.1	97.6	
**Respiratory difficulty**							
Yes	3	3.0	15.0	17	5.4	85.0	^a^*p* = 0.428
No	98	97.0	24.8	297	94.6	75.2	
**Skin redness**							
Yes	39	38.6	49.4	40	12.7	50.6	^a^*p* < 0.001
No	58	57.43	18.0	264	84.1	82.0	
Not known	4	4.0	28.6	10	3.2	71.4	
**Facial and/or neck swelling**							^a^*p* < 0.001
Yes	92	91.1	54.8	76	24.2	45.2	
No	9	8.9	3.7	235	74.8	96.3	
Not known			0.0	3	1.0	100.0	
**CRP level at hospital admission (mg/l)**^**d**^							^c^*p* = 0.906
Range	0–380			0–513			
Mean	149.5			146.0			
Median	128.5	IQR (86–214)		132.0	IQR (64.3–205)		
**White blood cell count at hospital admission (E**^**9**^**/l)**							^c^*p* = 0.025
Range	4.6–33.5			1.1–36.6			
Mean	13.5			14.9			
Median	12.9	IQR (10.2–15.5)		14.3	IQR (10.9–17.8)		
**Tympanic temperature**							^a^*p* < 0.001
< 38 °C	77	76.2	24.4	238	75.8	75.6	
≥38 °C	23	22.8	35.9	41	13.1	64.1	
Not known	1	1.0	2.8	35	11.1	97.2	
**Surgical intervention**							
Yes	97	96.0	33.6	192	61.1	66.4	^a^*p* < 0.001
No	4	4.0	3.2	122	38.9	96.8	
**Delay (days) from beginning of symptoms to hospital admission**							^c^*p* = 0.863
Range	0–22			0–44			
Mean	4.7			4.8			
Median	3.0	IQR (2–6)		4.0	IQR (2–6)		

*CRP* C-reactive protein, *IQR* interquartile range

^a^Fisher’s exact test

^b^Student’s *t*-test

^c^Mann-Whitney *U*

^d^CRP level was available in 100/101 of OIs and 258/314 of non-odontogenic infections

In all, 32 (7.7%) of all 415 patients required ICU care. Almost four out of five of these patients were men (78.1%). The difference was statistically significant compared with women (*p* = 0.024). Restricted mouth opening, facial or neck swelling (or both), higher tympanic temperature, and surgical intervention were associated with ICU care (*p* < 0.001) (Table 2). In all, 23.8% of OIs and 2.5% of ONPs were treated in ICU (*p* < 0.001). In total, 6 patients received tracheostomy for primary swelling of epiglottic area without ICU care.

**Table 2:** Associations between explanatory and predictor variables and need for intensive care unit stay

	Patients requiring ICU treatment			Patients without ICU treatment			
	n	%	% of patients	n	%	% of patients	***p-***value
**All**	32	7.7		383	92.5		
**Sex**							^a^*p* = 0.024
Male	25	78.1	10.3	218	56.9	89.7	
Female	7	21.9	4.1	165	43.1	95.9	
**Age (years)**							^b^*p* = 0.742
Range	22–81			16–95			
Mean	45.2			44.0			
Median	41.0			40.1			
**Smoking**							^a^*p* = 0.797
Yes	7	21.9	7.4	88	23.0	92.6	
No	9	28.1	6.4	131	34.2	93.6	
Not known	16	50.0	8.9	164	42.8	91.1	
**Heavy alcohol consumption**							^a^*p* = 0.264
Yes	2	6.3	15.4	11	2.9	84.6	
No	30	93.8	7.4	373	97.4	92.6	
**Immunodeficiency**							^a^*p* = 0.313
Yes	7	21.9	10.8	58	15.1	89.2	
No	25	78.1	7.1	325	84.9	92.9	
**Swallowing difficulty**							^a^*p* = 0.436
Yes	20	62.5	8.0	231	60.6	92.0	
No	9	28.1	5.8	147	38.4	94.2	
Not known	3	9.4	37.5	5	1.3	62.5	
**Restricted mouth opening**							^a^*p* < 0.001
Yes	26	81.3	16.8	129	33.7	83.2	
No	4	12.5	2.3	172	44.9	97.7	
Not known	2	6.3	2.4	82	21.4	97.6	
**Respiratory difficulty**							^a^*p* = 0.193
Yes	3	9.4	15.0	17	4.4	85.0	
No	29	90.6	7.3	366	95.6	92.7	
**Skin redness**							^a^*p* = 0.340
Yes	8	25.0	10.1	71	18.5	89.9	
No	22	68.8	6.8	300	78.3	93.2	
Not known	2	6.3	14.3	12	3.1	85.7	
**Facial and/or neck swelling**							^a^*p* < 0.001
Yes	27	84.4	16.1	141	36.8	83.9	
No	5	15.6	2.0	239	62.4	98.0	
Not known	0	0.0	0.0	3	0.8	100.0	
**CRP level at hospital admission (mg/l)**							^c^*p* = 0.095
Range	0–370			0–513			
Mean	191.0			142.7			
Median	209.0	IQR (115.3–238.0)		129.0	IQR (71.3–201.0)		
**White blood cell count at hospital admission (E**^**9**^**/l)**							^c^*p* = 0.867
Range	8.8–33.5			1.1–36.6			
Mean	15.5			14.3			
Median	13.8	IQR (11.5–17.7)		13.9	IQR (10.7–17.1)		
**Tympanic temperature**							^a^*p* < 0.001
< 38 °C	20	62.5	6.3	295	77.0	93.7	
≥ 38 °C	9	28.1	14.1	55	14.4	85.9	
Not known	3	9.4	8.3	33	8.6	91.7	
**Surgical intervention**							^a^*p* < 0.001
Yes	32	100	11.1	257	67.1	88.9	
No	0	0	0.0	126	32.9	100.0	
**Delay (days) from beginning of symptoms to hospital admission**							^c^*p* = 0.092
Range	1–22			0–44			
Mean	5.3			2.4			
Median	4.0	IQR (2–7)		2.0	IQR (2–6)		
**Odontogenic infection**							^a^*p* < 0.001
Yes	24	75	23.8	77	20.1	76.2	
No	8	25	2.5	306	79.9	97.5	
Peritonsillar and/or tonsillar and/or parapharyngeal infection^d^	5	15.6	2.4	205	53.5	97.6	
Sinusititis^e^	0	0.0	0.0	6	2.1	100.0	
Epiglottitis and/or supraglottitis^f^	1	3.1	2.4	41	10.7	97.6	
Sialadenitis^g^	0	0.0	0.0	31	8.1	100.0	
Other soft-tissue infection^h^	1	3.1	6.3	15	3.9	93.8	
Iatrogenic/trauma^i^	1	3.1	11.1	8	2.1	88.9	

International classification of disease (ICD-10)

*ICU* intensive care unit, *IQR* interquartile range

^a^Fisher’s exact test

^b^Student’s *t*-test

^c^Mann-Whitney *U*

^d^J02.0, J02.9, J03.0, J03.9, J35, J36, J39.0, J39.1

^e^J01.0, J01.4, J32.0, J32.2, J32.4

^f^J04.0. J05.1. J05.0. J38.4. J38.6

^g^ K11.2, K11.3, K11.5, K11.9

^h^ J34.8, K10.21, S02.67, S02.66, K12.2

^i^R60.9. J39., K12.18, L03.2, 12.11, K12.2

CRP level was available in 32/32 patients who needed ICU and in 327/384 for those who did not need ICU treatment

Logistic regression analyses revealed independent associations between sex and OIs with ICU care (Table 3). Women were significantly less likely to receive ICU care than men (odds ratio [OR] 0.351, 95% confidence interval [CI] 0.139–0.884; *p* = 0.026). Patients with OI were four times more likely to be treated in the ICU than patients with ONPs (95% OR 3.716, CI 1.189–11.618; *p* = 0.024).

**Table 3:** Binomial logistic regression model^a^ of selected variables of oro-naso-pharyngeal infection patients for intensive care unit stay

Categories	Coefficient	SE	*p*-value	OR	95% CI
Age	−0.005	0.011	0.633	0.995	0.972; 1.017
Sex	−1.047	0.471	0.026	0.351	0.139; 0.884
Immunosuppression	0.167	0.513	0.754	1.182	0.432; 3.229
Tympanic temperature	1.351	0.721	0.061	3.862	0.939; 15.880
Facial and or neck swelling	0.936	0.647	0.148	2.549	0.718; 9.057
Odontogenic infection	1.313	0.582	0.024	3.716	1.189; 11.618
Constant	−4.093	0.775	< 0.001	0.017	

*SE* standard error, *OR* odds ratio, *CI* confidence interval

^a^Hosmer and Lemeshow’s test for the model indicated good fit (χ^2^ = 6.994; df = 8; *P* = 0.543)

**Fig. 2:**
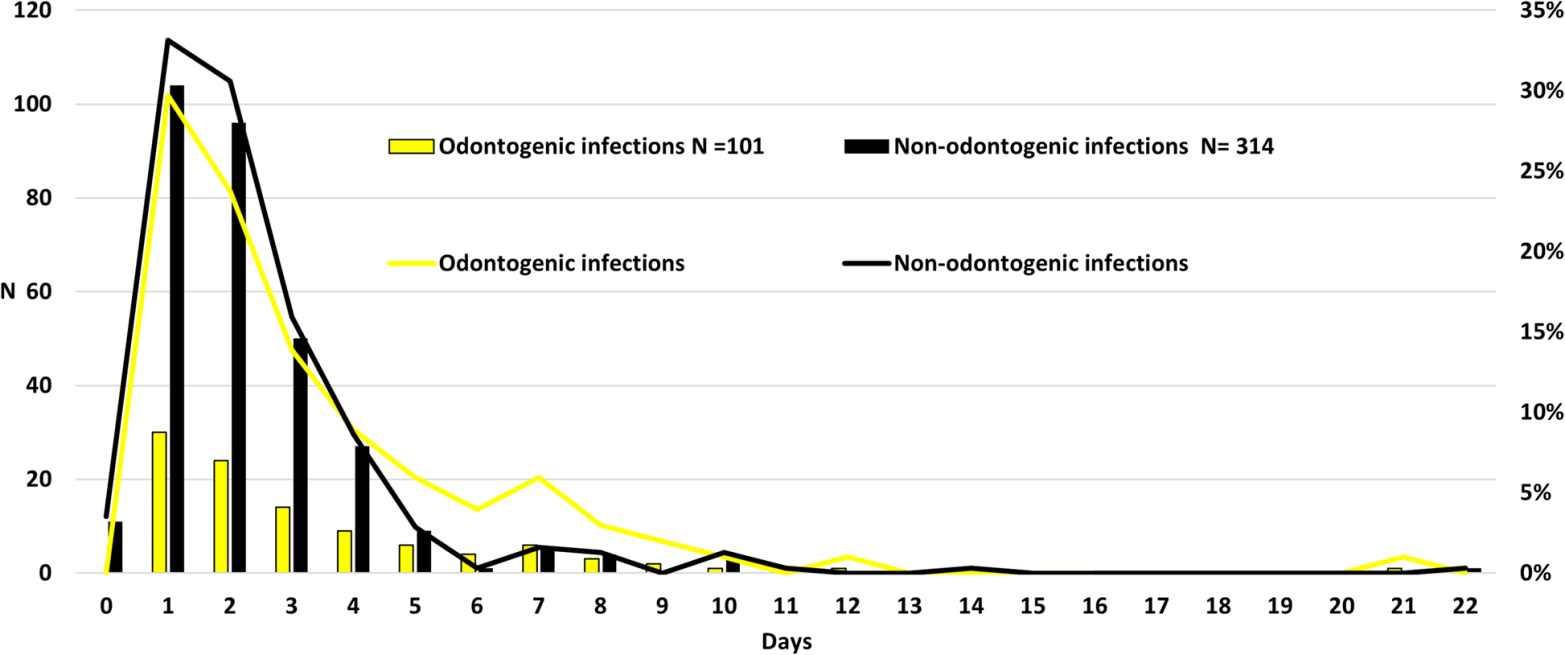
Length of hospital stay of oro-naso-pharyngeal infection types. The total length of hospital stay was significantly longer in patients with odontogenic infections than in patients with other oro-naso-pharyngeal infections (*p* = 0.017). The difference between infection groups in hospital stay is notable after the fourth treatment day

Length of hospital stay (LHOS) varied between less than 1 day to 21 days (mean 2.6, median 2). Only CRP level at hospital admission (*p* = 0.018) and facial or neck swelling (or both) (*p* = 0.006) were associated with LHOS ≥2 days (Table 4). However, when assessing the total hospital stay as a continuous variable, LHOS was significantly longer in patients with OI than in those with ONPs (*p* = 0.017) (Fig. 2).

**Table 4:** Associations between explanatory and predictor variables and duration of hospital stay

	Hospital stay < 2 days			Hospital stay ≥ 2 days			
	n	%	% of patients	n	%	% of patients	***p***-value
**All**	145	34.9		270	65.1		
**Sex**							^a^*p* = 0.174
Male	78	53.8	32.1	165	61.1	67.9	
Female	67	46.2	39.0	105	38.9	61.0	
**Age (years)**							^b^*p* = 0.066
Range	16–89			16–95			
Mean	41.6			45.5			
Median	36.8			42.4			
**Smoking**							
Yes	33	22.1	34.7	62	22.6	65.3	^a^*p* = 0.584
No	54	37.2	38.6	86	31.9	61.4	
Not known	58	40.0	32.2	122	45.6	67.8	
**Heavy alcohol consumption**							
Yes	2	1.4	15.4	11	4.1	84.6	^a^*p* = 0.235
No	143	98.6	35.6	259	95.9	64.4	
**Immunodeficiency**							
Yes	16	11.0	24.6	49	18.1	75.4	^a^*p* = 0.066
No	129	89.0	36.9	221	81.9	63.1	
**Swallowing difficulty**							
Yes	86	59.3	34.3	165	61.1	65.7	^a^*p* = 0.915
No	55	37.9	35.3	101	37.4	64.7	
Not known	4	2.8	50.0	4	1.5	50.0	
**Restricted mouth opening**							
Yes	46	31.7	29.7	109	40.4	70.3	^a^*p* = 0.065
No	71	49.0	40.3	105	38.9	59.7	
Not known	28	19.3	33.3	56	20.7	66.7	
**Respiratory difficulty**							
Yes	3	2.1	15.0	17	6.3	85.0	^a^*p* = 0.058
No	142	97.9	35.9	253	93.7	64.1	
**Facial skin redness**							
Yes	24	16.6	30.4	55	20.4	69.6	^a^*p* = 0.297
No	119	82.1	37.0	203	75.2	63.0	
Not known	2	1.4	14.3	12	4.4	85.7	
**Facial and/or neck swelling**							
Yes	46	31.7	27.4	122	45.2	72.6	^a^*p* = 0.006
No	99	68.3	40.6	145	53.7	59.4	
Not known	0	0	0.0	3	1.1	100.0	
**CRP level at hospital admission (mg/l)**							
Range	0–504			0–513			^c^*p* = 0.018
Mean	127.8			156.5			
Median	109.0	IQR (56–175.5)		143.0	IQR (80.3–216)		
**White blood cell count at hospital admission (E**^**9**^**/l)**							
Range	1.1–36.6			3.6–33.7			^c^*p* = 0.109
Mean	13.6			14.8			
Median	13.0	IQR (10.5–15.8)		14.2	IQR (11–17.5)		
**Tympanic temperature**							
< 38 °C	16	11.0	25.0	48	17.8	75.0	^a^*p* = 0.180
≥ 38 °C	116	80	36.8	199	73.7	63.2	
Not known	13	9.0	36.1	23	8.5	63.9	
**Surgical intervention**							
Yes	97	66.9	33.6	192	71.1	66.4	^a^*p* = 0.373
Additional tracheostomy	0	0	0.0	8	3.0	100.0	
No	48	33.1	38.1	78	28.9	61.9	
**Delay (days) from beginning of symptoms to hospital admission**							
Range	0–21			0–44			^c^*p* = 0.893
Mean	4.6			4.8			
Median	3.0	IQR (2–6)		4.0	IQR (2–6)		
**Odontogenic infection**							
Yes	30	20.7	29.7	71	26.3	70.3	^a^*p* = 0.231
No	115	79.3	36.6	199	73.7	63.4	
Peritonsillar and/or tonsillar infection and/or parapharyngeal infection	92	63.4	43.8	118	43.7	56.2	
Sinusititis	1	0.7	16.7	5	1.9	83.3	
Epiglottitis and/or supraglottitis	8	5.5	19.0	34	12.6	81.0	
Sialadenitis	9	6.2	29.0	22	8.1	71.0	
Iatrogenic/trauma	2	1.4	22.2	7	2.6	77.8	
Other soft-tissue infection	3	2.1	18.8	13	4.8	81.3	

*CRP* C-reactive protein, *IQR* interquartile range

^a^Fisher’s exact test

^b^Student’s *t*-test

^c^Mann-Whitney *U*

CRP level was available in 118/145 of patients with hospital stay < 2 days and in 240/270 of patients with hospital stay ≥2 days

## Discussion

The purpose of the present study was to clarify differences in infection characteristics between patients hospitalized with OI or another bacterial ONP infection. The specific aim was to evaluate clinical infection variables and infection severity according to infection aetiology and to clarify the features of OIs. A quarter of all ONP infections were OIs (Fig. 1), and dental origin was the second most common aetiology after pharyngeal region infections. The hypothesis was that OIs have special features that differ from other ONP infections, and this was confirmed. Hospitalized patients with OI more often had restricted mouth opening, redness of the facial skin, and facial or neck swelling (or both). LHOS was significantly longer in patients with OI (Fig. 2) and significantly more often required ICU care (Table 3) than ONPs. Thus, OIs are a clinically notable cause for hospitalization when all ONP infections are considered.

The spectrum of symptoms and findings in ONP infections is wide given the differences in aetiology. Features reported most often include dysphagia, fever, malaise, odynophagia, ipsilateral otalgia, severe sore throat, cervical lymphadenitis, trismus, and swelling of neck, face, tongue base, and oral cavity [6, 12–14]. Previously, studies that clarified features of different ONP infections focused mainly on life-threatening conditions, such mediastinitis, necrotizing fasciitis, and Lemierre’s syndrome [15]. For example, mediastinal spread occurs more commonly in non-odontogenic deep-neck infections [8, 16] than those of dental origin. The present study focused more widely on clinical findings at the time of hospital admission. Typical features of OIs were restricted mouth opening, redness of facial skin, and facial or neck swelling (or both). Thus, it is essential to consider odontogenic aetiology if these findings occur, to achieve optimal treatment. OIs are almost always treated by surgical intervention, which includes abscess drainage and removal of the focus tooth.

In contrast to previous studies focused on hospital stay [6, 12], patients with OIs had longer LHOS than patients with other ONPs. We compared OIs to all bacterial infections of ONP region, which may be responsible for this difference. However, OIs more often required ICU treatment, as 75% of all patients that were treated in ICU had an OI. Airway management and mechanical ventilation were the main reasons for ICU treatment. However, 6 patients with epiglottitis received tracheostomy and were treated at the ward. Thus, ICU care for treatment of a compromised airway may influence the results. On the other hand, the entire LHOS was significantly longer in OI patients. It should be emphasized that the need for hospital care and most OIs in general can be prevented by improving preventive care (i.e., regular dental care and effective treatment of an incipient infection). In addition, there should be a greater emphasis on earlier identification of these infections, as both medical and dental professionals have difficulties in detecting OIs [17].

A peritonsillar abscess is the most common otorhinolaryngological infection requiring hospitalization [18]. The annual incidence of peritonsillar abscess is 9/100000/y [19]. Overall, oropharyngeal infections, including peritonsillar infections, were the most common infections of all hospitalized patients according to our results. However, only 5 of these required prolonged intubations. Patient characteristics and clinical findings differed when compared with OIs. Patients in this group were younger than those with OIs (mean 42 years, median 38 years), had on average slightly higher infection parameters, and more often had difficulty swallowing. On the other hand, patients with OIs more often had facial swelling, restricted mouth opening, and redness of the skin. Respiratory difficulties also occurred more often among patients with OIs. Our results may assist clinicians in differential diagnostics between OIs. Patients with infections of the oropharyngeal region should be referred to the most suitable treatment facility.

Among other ONP infections, epiglottitis was the third most common group in this study. Acute epiglottitis in adults have similar symptoms as other ONP infections [20]. The dominant symptom in our study was swallowing difficulty (observed in two-thirds of epiglottitis patients), which is an essential sign of dyspnoea with laryngeal oedema, which may lead to sudden upper-airway obstruction [15, 21]. All other clinical parameters in epiglottitis patients were clearly more uncommon. Infections that originate from salivary stone and obstruction and other conditions on the mucosal surface of the upper aerodigestive track can also lead to hospitalization and severe infections. However, the present study showed that these aetiologies are rare, especially when considering the need for ICU care. Only one infected mandibular fracture and tongue abscess required ICU care. The remaining patients all had OIs, epiglottitis, or peritonsillar or parapharyngeal abscesses.

The limited accuracy of some variables and particularly clinical findings may be due to the retrospective study design. Additionally, the number of patients in rarer ONP subgroups remained low; thus, detailed analyses for these infection types were not conducted. A prospective study design would be beneficial to clarify differential diagnostics in more detail.

## Conclusion

The present study showed that of ONP infections, especially OIs and oropharyngeal infections are resource-intensive for hospitals. Infections of dental origin more frequently require intensive care and a longer hospital stay than other types of ONP infections. Severe OIs have different clinical features than other ONP infections, which should be emphasized to achieve early and prompt diagnosis and treatment.

## Data Availability

The datasets used and analysed during the current study are available from the corresponding author on reasonable request.

## Abbreviations

ONP: Oro-naso-pharyngeal
OI: Odontogenic infection
ICU: Intensive care unit
LHOS: Length of hospital stay
CRP: C-reactive protein
